# Adequate lymph node dissection is essential for accurate nodal staging in intrahepatic cholangiocarcinoma: A population‐based study

**DOI:** 10.1002/cam4.5620

**Published:** 2023-01-16

**Authors:** Jiang Zhu, Chang Liu, Hui Li, Haoyu Ren, Yunshi Cai, Tian Lan, Hong Wu

**Affiliations:** ^1^ Liver Transplantation Center, West China Hospital Sichuan University Chengdu China; ^2^ Department of Liver Surgery, West China Hospital Sichuan University Chengdu China; ^3^ Department of Hepatopancreatobiliary Minimal Invasive Surgery Chengdu ShangJin NanFu Hospital Chengdu China; ^4^ Department of Hepatobiliary Pancreatic Tumor Center Chongqing Key Laboratory of Translational Research for Cancer Metastasis and Individualized Treatment Chongqing University Cancer Hospital Chongqing China; ^5^ Department of Breast and Thyroid Surgery The Second Affiliated Hospital of Chongqing Medical University Chongqing China; ^6^ Department of General, Visceral and Transplantation Surgery, University Hospital LMU Munich Munich Germany

**Keywords:** intrahepatic cholangiocarcinoma, lymph node dissection, nodal stage, number of lymph node metastasis, prognosis

## Abstract

**Purpose:**

To comprehensively investigate the implications of lymph node dissection (LND) and the prognostic impact of the number of lymph node (LN) metastases on survival in intrahepatic cholangiocarcinoma (ICC) using a large‐scale study.

**Methods:**

Patients who underwent surgical resection for ICC between 2004 and 2018 were identified from the Surveillance, Epidemiology, and End Results (SEER) registries. The Kaplan–Meier and log‐rank tests were used to compare cancer‐specific survival (CSS) and overall survival (OS) between different groups. Propensity score matching (PSM) and subgroup analyses were performed to balance potential confounding factors. A multivariate Cox proportional hazards regression model was used to identify prognostic factors of survival outcomes. Restricted cubic splines fitted in the Cox proportional hazard regression models were also conducted to examine associations between continuous variables and outcomes.

**Results:**

In all, 1028 patients were enrolled. There were 652 (63.4%) patients undergoing LND, with lymph node metastasis (LNM) confirmed in 212 (32.5%) cases. Patients receiving LND did not show better survival outcomes than those receiving non‐LND (NLND). We divided the LND group into two subgroups: patients with LNM (+) and those without LNM (−). Among these three groups, patients with LNM experienced the worst CSS and OS, while NLND patients had similar survival times to LNM (−) patients. Restricted cubic spline analysis indicated that an increased number of LNM was associated with a decreased chance of survival (*p* < 0.001). Patients who received LND were further categorized as having no nodal metastasis (N0), 1–2 LNM (N1), or ≥3 LNM (N2) according to the number of LNM. The Kaplan–Meier curves showed that the mortality risk of patients with N0, N1, and N2 disease (median CSS, N0 50.0 vs. N1 22.0 vs. N2 14.0 months; median OS, N0 46.0 vs. N1 21.0 vs. N2 14.0 months, all *p* < 0.01) increased significantly, except for patients who had <6 LNs harvested. On multivariable survival analysis, a higher nodal stage (N1 vs. N0: CSS, hazard ratio [HR] 2.135, 95% CI 1.636–2.788, *p* < 0.001; OS, HR 2.100, 95% CI 1.624–2.717, *p* < 0.001; N2 vs. N0: CSS, HR 4.027, 95% CI 2.791–5.811, *p* < 0.001; OS, HR 3.678, 95% CI 2.561–5.282, *p* < 0.001) was an independent prognostic risk factor for survival.

**Conclusions:**

Despite the lack of a clear survival benefit of LND in patients with ICC, a significant positive association between the number of LNM and poor outcomes was observed. We still suggest adequate LND by examining at least six LNs to ensure precise staging. On this basis, the recently proposed nodal classification of N0, N1, and N2 stages may also allow better prognostic stratification of ICC patients.

## Graphical abstract

Regarding ICC, the eighth edition of the American Joint Committee on Cancer (AJCC) staging system simply classified the N stage as N0 and N1 on the basis of LN status. The implications of lymph node dissection (LND) and the prognostic impact of the number of lymph node metastases on survival in intrahepatic cholangiocarcinoma (ICC) remain unclear. By analyzing a large cohort of more than 1000 resected ICC patients, the present study showed that despite the lack of a clear survival benefit of LND in patients with ICC, a Significant positive association between the number of LNM and poor outcomes was observed. We still suggest adequate LND by examining at least 6 lymph nodes to ensure precise staging. On this basis, the recently proposed nodal classification of N0, N1, and N2 stages may also allow better prognostic stratification of ICC patients.

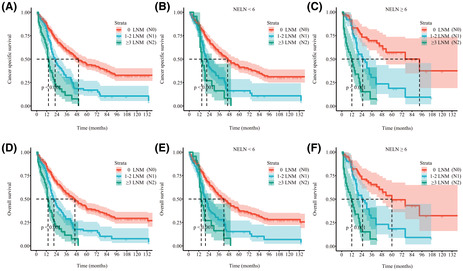

## 1 INTRODUCTION

Intrahepatic cholangiocarcinoma (ICC), originating from intrahepatic biliary epithelial cells, is a highly lethal disease with a grim prognosis.^1^, ^2^, ^3^ To date, although the therapeutic effect is not satisfactory, surgical resection remains the only chance of a cure due to limited treatment options.^4^, ^5^, ^6^ It is worth mentioning that the role of lymph node dissection (LND) is among the most contentious in the field of surgical therapy of ICC.^7^, ^8^ Many surgeons hold conservative views on the value of LND, with several studies reporting that LND does not improve survival outcomes but possibly increases the extra risk of complications as well as operative time.^9^, ^10^, ^11^, ^12^ However, it is clear that lymph node metastasis (LNM) in ICC can be found in up to 25%–60% of patients with ICC after the operation.^13^, ^14^, ^15^ Thus, adequate LND is crucial to ensure oncological radicality; otherwise, disease recurrence in residual metastatic lymph nodes (LNs) may lead to extrahepatic biliary obstruction or make it unresectable. Furthermore, LND is essential to provide precise pathological staging to guide decisions around postoperative adjuvant treatment for clinicians and inform patients about prognosis.^12^, ^16^, ^17^ Currently, worldwide mainstream official practice guidelines, almost without exception, recommend considering routine lymphadenectomy for patients at the time of ICC resection.^18^ In addition, for many cancers, such as perihilar and distal cholangiocarcinoma, the nodal (N) classification system was subdivided into no nodal metastasis (N0), 1–3 LNM (N1), and ≥4 LNM (N2) according to the number of metastatic LNs and showed good performance in prognostic stratification.^19^, ^20^ Regarding ICC, the eighth edition of the American Joint Committee on Cancer (AJCC) staging system simply classified the N stage as N0 and N1 on the basis of LN status.^21^ In the era of personalized medicine, this simplistic binary classification for N staging bears limited prognostic value to guide clinical practice. However, data focusing on the association between the number of LNM and the prognosis of ICC are limited. Hence, the objective of this study was to evaluate the clinical implications of LND. More importantly, we especially sought to investigate the association of the number of LNM with the survival of patients after surgical resection for ICC.

## 2 MATERIALS AND METHODS

### 2.1 Data source and patient selection

We identified patients diagnosed with ICC from January 1, 2004, to December 31, 2018, from the Surveillance, Epidemiology, and End Results (SEER) database through SEER*Stat software (v.8.3.9). The inclusion criteria included the following: (1) the primary site code for liver (C22.0) and intrahepatic bile duct (C22.1) with histology code 8160/3 (cholangiocarcinoma) based on the third edition International Classification of Disease for Oncology (ICD‐O‐3); (2) patients aged 18–80 years old with the first and only one primary malignancy; (3) patients confirmed by histology who received cancer‐directed surgery (surgery of primary site codes: 20–90); (4) patients with known information on LND status, radiation, tumor size, tumor (T) stage, and without distant metastasis; and (5) patients who survived more than 1 month after surgery. Patients with unknown survival data and those who underwent LND but without information on LN status were excluded. The following variables were collected from the database: age, year of diagnosis, sex, race, marital status, tumor size, distant metastasis, tumor grade, the extent of surgery, chemotherapy status, radiation therapy status, follow‐up outcome, survival months, the scope of LND, LN status, number of examined lymph nodes (NELN), and metastatic LNs. The T‐stage of patients was reclassified according to the eighth edition of the AJCC staging system for ICC. Cancer‐specific survival (CSS) was calculated from the date of diagnosis to the date of death attributed to ICC. Overall survival (OS) was defined as the period from the date of diagnosis to the date of death.

### 2.2 Statistical analysis

Categorical variables were compared by the Fisher's exact tests across groups. Propensity‐score matching (PSM) was performed by using the 1:1 nearest neighbor matching method with a caliper of 0.05 on the propensity scale to balance confounding factors between the LND group and the non‐LND group (NLND). The matching variables included sex, age, year of diagnosis, race, marital status, tumor grade, T‐stage, tumor size, and treatment options (radiation, chemotherapy, surgery). The CSS and OS of ICC patients were estimated with the Kaplan–Meier method and then compared using log‐rank tests. Univariate and multivariate Cox proportional hazard regression analyses were used to evaluate the effect of each factor on prognosis. The outcome risks of CSS and OS were expressed as hazard ratios (HR) with corresponding 95% confidence intervals (CI). Subgroup analyses were also performed to clarify the effect of LND on the outcome. Among patients who underwent LND, we used restricted cubic splines (RCS) with four knots based on Cox proportional hazards regression models to flexibly model the association of the number of LNM with CSS and OS. A *p* value <0.05 in a two‐tailed test was considered statistically significant. All analyses were conducted using R software version 4.0.2 (R Foundation for Statistical Computing).

## 3 RESULTS

### 3.1 Demographic and clinicopathological characteristics

There were 1028 patients with ICC who underwent curative resection in the SEER database from 2004 to 2018 and met the inclusion and exclusion criteria (Figure S1). Of those patients, 652 (63.4%) underwent lymphadenectomy. The proportions of patients with LND increased from 2004–2008 to 2014–2018, and LND was performed more often in younger patients. Additionally, patients with LND were more likely to receive aggressive treatment regimens (major liver resection, chemotherapy, and radiotherapy) than patients without LND. PSM was utilized to adjust for imbalanced covariates between the LND and NLND groups (Figure S2). A total of 355 patients in each group were matched. After matching, baseline characteristics were comparable. The clinicopathologic features of patients who underwent lymphadenectomy versus those who did not before and after PSM are detailed in Table 1.

**TABLE 1:** Clinicopathologic characteristic of patients undergoing surgery for intrahepatic cholangiocarcinoma before and after PSM

Characteristics	Before PSM (*N* = 1028)				After PSM (*N* = 710)			
	Overall *N* = 1028 (%)	NLND *N* = 376 (%)	LND *N* = 652 (%)	*p* value	Overall *N* = 710 (%)	NLND *N* = 355 (%)	LND *N* = 355 (%)	*p* value
Age (year)				<0.001				0.083
≤60	438 (42.6)	135 (35.4)	305 (46.8)		277 (39.0)	129 (36.3)	148 (41.7)	
>60	590 (57.4)	243 (64.6)	347 (53.2)		443 (61.0)	226 (63.7)	207 (58.3)	
Sex				1.00				1.00
Male	481 (46.8)	176 (46.8)	305 (46.8)		333 (46.9)	166 (46.8)	167 (47.0)	
Female	547 (53.2)	200 (53.2)	347 (53.2)		377 (53.1)	189 (53.2)	188 (53.0)	
Race				0.141				0.092
White	805 (78.3)	282 (75.0)	523 (80.2)		549 (77.3)	265 (74.6)	284 (80.0)	
Asian	135 (13.1)	58 (15.4)	77 (11.8)		96 (13.5)	57 (16.1)	39 (11.0)	
Black	86 (8.4)	36 (9.6)	50 (7.7)		63 (8.9)	33 (9.3)	30 (8.5)	
Other	2 (0.2)	0 (0)	2 (0.3)		2 (0.3)	0 (0)	2 (0.6)	
Marital status				0.469				0.297
Married	650 (63.2)	240 (63.8)	410 (62.9)		443 (62.4)	231 (65.1)	212 (59.7)	
Single	156 (15.2)	51 (13.6)	105 (16.1)		107 (15.1)	47(13.2)	60 (16.9)	
DSW	178 (17.3)	65 (17.3)	113 (17.3)		130 (18.3)	60 (16.9)	70 (19.7)	
Unknown	44 (4.3)	20 (5.3)	24 (3.7)		30 (4.2)	17 (4.8)	13 (3.7)	
Year of diagnosis				0.019				0.343
2004–2008	204 (19.8)	81 (21.5)	123 (18.9)		133 (18.7)	69 (19.4)	64 (18.0)	
2009–2013	325 (31.6)	134 (35.6)	191 (29.3)		275 (38.7)	128 (36.1)	147 (41.4)	
2014–2018	499 (48.5)	161 (42.8)	338 (51.8)		302 (42.5)	158 (44.5)	144 (40.6)	
Tumor size (mm)								0.258
≤20	73 (7.1)	22 (5.8)	51 (7.8)	0.305	53 (7.5)	21 (5.9)	32 (9.0)	
20–50	401 (39.0)	156 (41.5)	245 (37.6)		144 (40.8)	144 (40.6)	146 (41.1)	
>50	554 (53.9)	198 (52.7)	356 (54.6)		190 (51.7)	190 (53.5)	177 (49.9)	
Grade				0.642				0.844
I	96 (9.3)	39 (10.4)	57 (8.8)		68 (9.6)	35 (9.9)	33 (9.3)	
II	523 (50.9)	187 (49.7)	336 (51.5)		367 (51.7)	178 (50.1)	189 (53.2)	
III/IV	289 (28.1)	102 (27.1)	187 (28.7)		185 (26.1)	97 (27.3)	88 (24.8)	
Unknown	120 (11.7)	48 (12.8)	72 (11.0)		90 (12.7)	45 (12.7)	45 (12.7)	
AJCC T‐stage				<0.001				0.974
T1a	240 (23.3)	111 (29.5)	129 (19.8)		199 (28.0)	98 (27.6)	101 (28.5)	
T1b	192 (18.7)	83 (22.1)	109 (16.7)		157 (22.1)	77 (21.7)	80 (22.5)	
T2	446 (43.4)	153 (40.7)	293 (44.9)		296 (41.7)	151 (42.5)	145 (40.8)	
T3/4	150 (14.6)	29 (7.7)	121 (18.6)		58 (8.2)	29 (8.2)	29 (8.2)	
Surgery				0.010				0.095
Major resection	242 (23.5)	73 (19.4)	169 (25.9)		126 (17.7)	72 (20.3)	54 (15.2)	
Minor resection	786 (76.5)	303 (80.6)	483 (74.1)		584 (82.3)	283 (79.7)	301 (84.8)	
Chemotherapy				<0.001				0.448
Yes	523 (50.9)	146 (38.8)	377 (57.8)		303 (42.7)	146 (41.1)	157 (44.2)	
No	505 (49.1)	230 (61.2)	275 (42.2)		407 (57.3)	209 (58.9)	198 (55.8)	
Radiation				0.015				0.821
Yes	166 (16.1)	48 (12.8)	118 (18.1)		89 (12.5)	46 (13.0)	43 (12.1)	
No	862 (83.9)	328 (87.2)	534 (81.9)		621 (87.5)	309 (87.0)	312 (87.9)	

Abbreviations: AJCC, American Joint Committee on Cancer; DSW, divorced/separated/widowed; LND, lymph node dissection; NLND, non‐LND; PSM, propensity score matching.

### 3.2 Prognostic impact of LND

**FIGURE 1:**
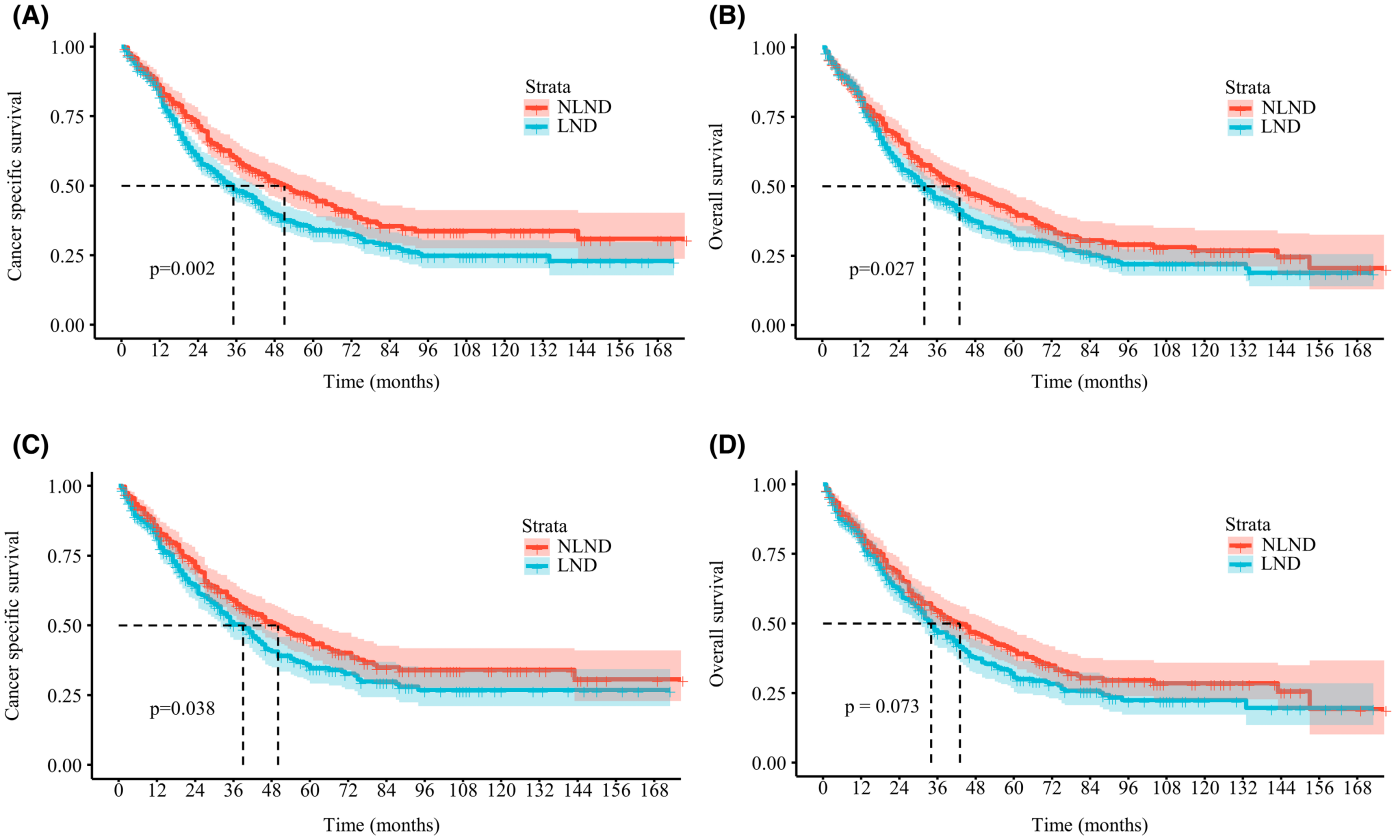
Kaplan–Meier curves of patients undergoing lymph node dissection (LND) or not for intrahepatic cholangiocarcinoma. Cancer‐specific survival (CSS) (A) and overall survival (OS) (B) of patients before propensity score matching (PSM). CSS (C) and OS (D) of patients after PSM. NLND, non‐LND

**FIGURE 2:**
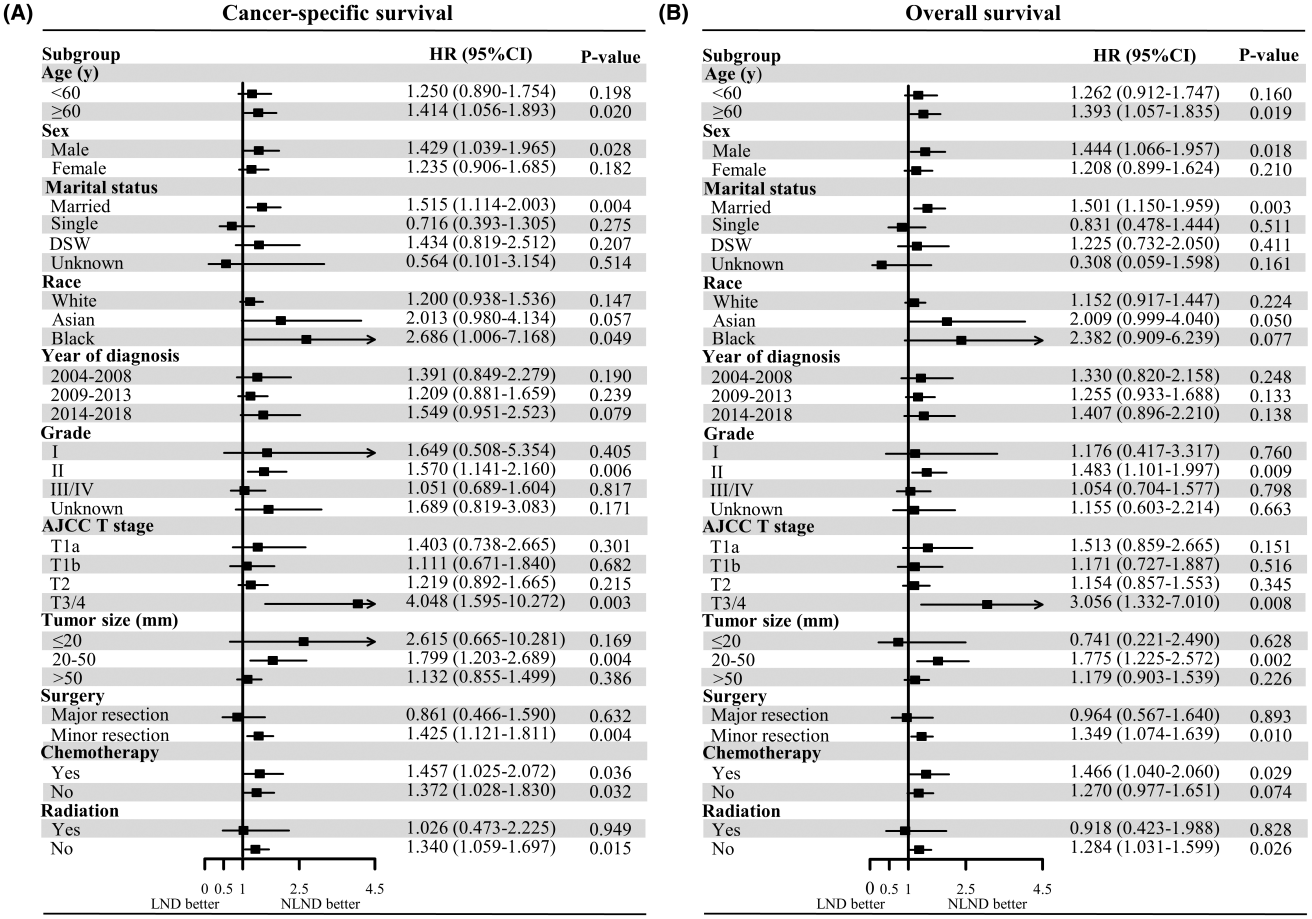
Forest plot of multivariable Cox proportional hazard regression analysis for the effect of LND on CSS (A) and OS (B) by subgroup analyses after PSM. AJCC, American Joint Committee on Cancer; CSS, cancer‐specific survival; DSW, divorced/separated/widowed; LND, lymph node dissection; NLND, non‐LND; OS, overall survival; PSM, propensity score matching

**FIGURE 3:**
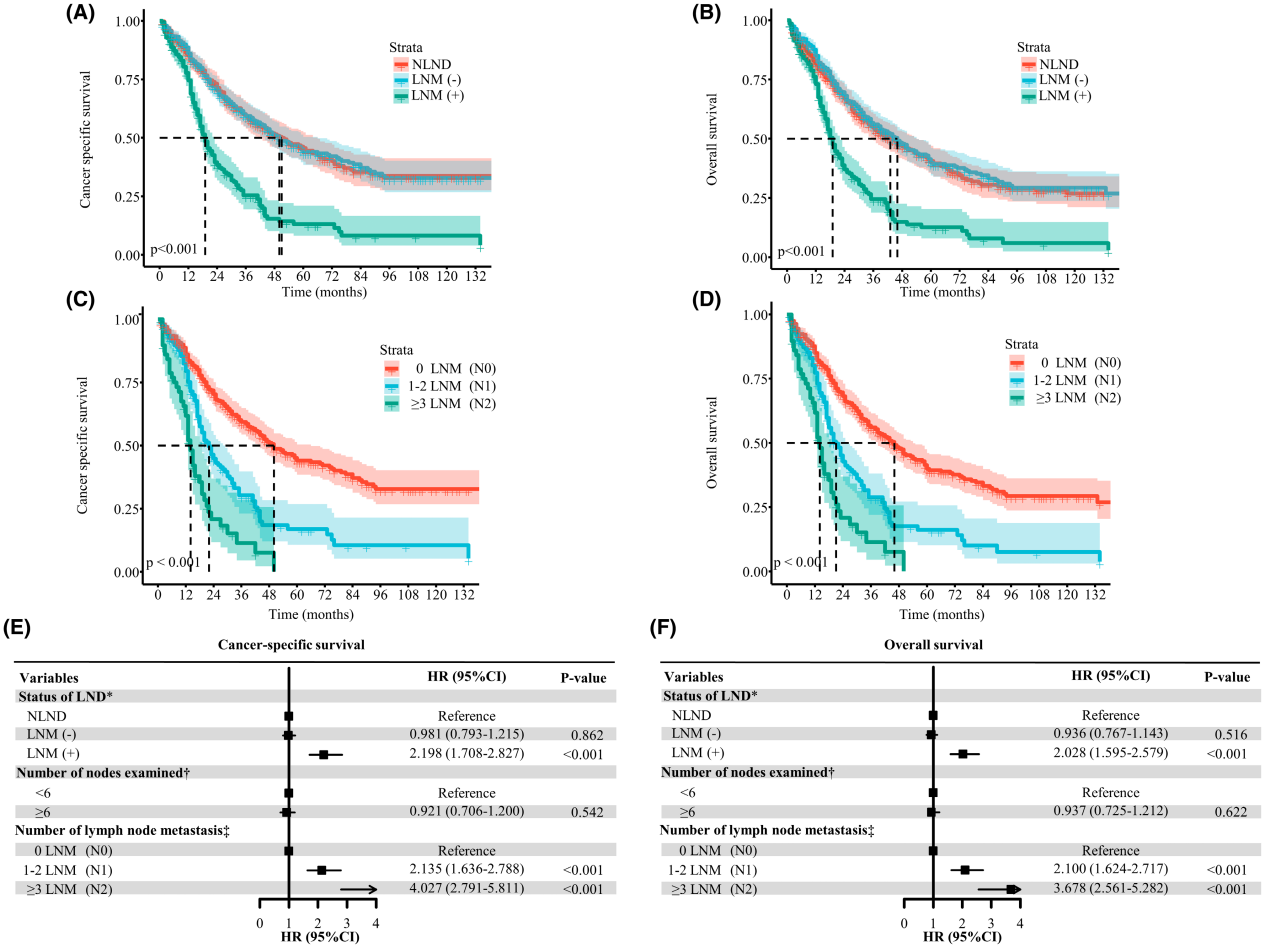
Kaplan–Meier curves of CSS (A) and OS (B) among NLND patients, LND patients with lymph node metastasis (LNM+), and no lymph node metastasis (LNM−). Kaplan–Meier curves of CSS (C) and OS (D) among patients with no nodal disease, 1–2 lymph node metastasis (LNM) and ≥3 LNM. Multivariable‐adjusted HR (95% CI) for CSS (E) and OS (F). *Adjusted for age, sex, race, marital status, grade, tumor size, AJCC stage, surgery, chemotherapy, and radiation. †Adjusted for age, sex, race, marital status, grade, tumor size, AJCC stage, surgery, chemotherapy, radiation, and lymph node status. ‡Adjusted for age, sex, race, marital status, grade, tumor size, AJCC stage, surgery, chemotherapy, radiation, and number of examined lymph nodes. AJCC, American Joint Committee on Cancer; CI, confidence interval; CSS, cancer‐specific survival; HR, hazard ratio; LND, lymph node dissection; LNM, lymph node metastasis; NLND, non‐LND; OS, overall survival

**FIGURE 4:**
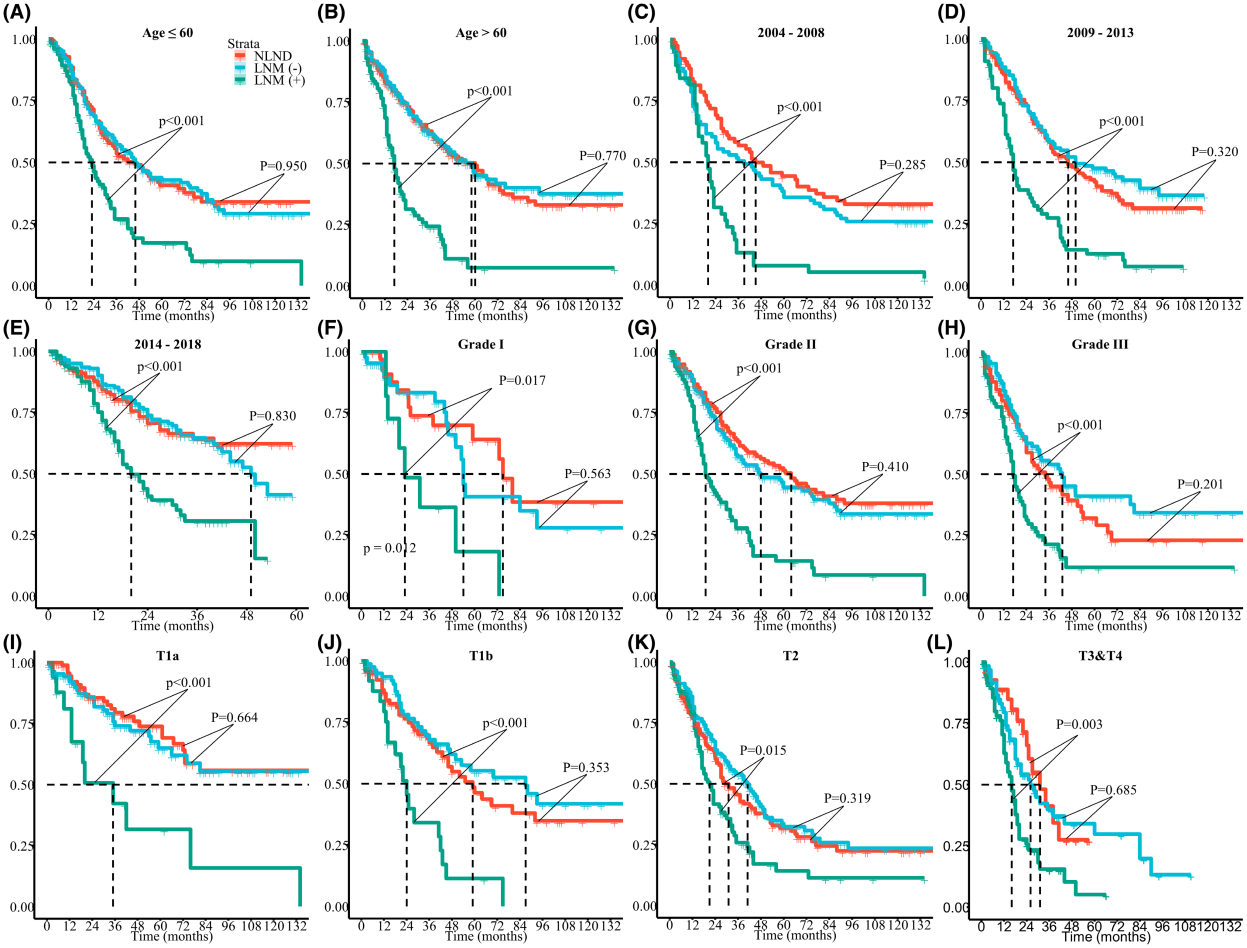
Kaplan–Meier curves of CSS among NLND, LNM (+), and LNM (−) groups as stratified by age (A,B), year of diagnosis (C–E), grade (F–H), and AJCC T‐stage (I–L). AJCC, American Joint Committee on Cancer; CSS, cancer‐specific survival; LND, lymph node dissection; NLND, non‐LND; LNM, lymph node metastasis

The 5‐year CSS and OS rates of the overall cohort were 38.4% (95% CI, 34.8%–42.3%) and 34.8% (95% CI, 31.4%–38.5%), respectively. The median follow‐up period was 35 months (interquartile range [IQR]: 16–94 months). Interestingly, in the Kaplan–Meier analysis, patients with LND had poorer CSS (5‐year CSS, 34.1% vs. 45.7%; *p* = 0.002) and OS (5‐year OS, 31.2% vs. 40.6% *p* = 0.027) than patients without LND (Figure 1A,B). Similarly, lymphadenectomy was associated with worse survival after PSM (Figure 1C,D). On multivariate Cox proportional hazards regression analysis, the patients with LND had an increased HR of death when compared to patients without LND regardless of matching (Tables 2 and 3). In the matched cohort, LND was independently associated with worse survival across most baseline subgroups (Figure 2A,B). We sought to determine whether the above results could be attributable to intrinsic biases of the baseline characteristics between the LND and NLND groups. Therefore, we next split the LND group into two subgroups, patients with LNM (+) and those without LNM (−), and their survival outcomes were compared with those of the NLND group. LNM was identified in 212 (32.5%) patients. The characteristics of the three groups are summarized in Table 4. Of note, among these three groups, the LNM (+) group (median time, LNM (+) 19 vs. NLND 50 months, *p* < 0.001) had the worst CSS, followed by LNM (−) patients and NLND patients (median time, LNM (−) 51 vs. NLND 50 months, *p* = 0.920) (Figure 3A). Likewise, patients with LNM had the shortest OS (median time, LNM (+) 19 vs. NLND 43 months, *p* < 0.001), while NLND patients showed similar survival time to LNM (−) patients (median time, LNM (−) 46 vs. NLND 43 months, *p* = 0.450) (Figure 3B). In subgroup analyses, we again observed no significant differences in CSS (Figure 4) or OS (Figure S3) among the NLND group and LNM (−) groups with respect to different years of diagnosis, ages, tumor grade, and T stages (all *p* > 0.05). Nevertheless, the LNM (+) group suffered significantly worse survival than the other two groups (all *p* < 0.05). Consistent with univariate analysis, multivariable analysis confirmed that as compared with the NLND group, the survival outcomes of the LNM (−) subgroup were comparable (*p* > 0.05), whereas the LNM (+) subgroup was associated with significantly poorer CSS (HR: 2.198, 95% CI 1.708–2.827, *p* < 0.001) and OS (HR: 2.028, 95% CI 1.595–2.579, *p* < 0.001) (Figure 3E,F).

**TABLE 2:** Univariate and multivariate Cox proportional hazard regression analysis of CSS and OS before PSM

Variables	CSS				OS			
	Univariate analysis		Multivariate analysis		Univariate analysis		Multivariate analysis	
	HR (95% CI)	*p* value	HR (95% CI)	*p* value	HR (95% CI)	*p* value	HR (95% CI)	*p* value
Sex (male vs. female)	1.210 (0.979–1.497)	0.078	1.378 (1.094–1.736)	0.006	1.269 (1.040–1.548)	0.019	1.420 (1.143–1.764)	0.002
Age (year) (>60 vs. ≤60)	1.039 (0.838–1.288)	0.730	1.056 (0.842–1.324)	0.638	1.130 (0.922–1.385)	0.238	1.126 (0.909–1.394)	0.278
Race								
White	Reference		Reference		Reference		Reference	
Asian	0.936 (0.684–1.281)	0.680	0.996 (0.721–1.377)	0.982	0.834 (0.616–1.131)	0.244	0.877 (0.641–1.199)	0.412
Black	1.210 (0.838–1.747)	0.308	1.204 (0.824–1.760)	0.337	1.074 (0.750–1.538)	0.698	1.045 (0.722–1.514)	0.815
Other	0.677 (0.095–4.827)	0.697	0.640 (0.088–4.660)	0.659	0.576 (0.081–4.105)	0.582	0.613 (0.084–4.449)	0.628
Marital status								
Married	Reference		Reference		Reference		Reference	
Single	0.925 (0.679–1.258)	0.618	0.891 (0.649–1.222)	0.473	0.931 (0.698–1.243)	0.628	0.900 (0.669–1.211)	0.487
DSW	1.140 (0.861–1.510)	0.361	1.176 (0.862–1.603)	0.307	1.166 (0.898–1.516)	0.249	1.182 (0.886–1.578)	0.255
Unknown	1.404 (0.844–2.334)	0.191	1.522 (0.904–2.561)	0.114	1.394 (0.863–2.252)	0.174	1.518 (0.929–2.480)	0.096
Tumor size (cm)								
≤20	Reference		Reference		Reference		Reference	
20–50	1.282 (0.779–2.108)	0.328	1.473 (0.881–2.464)	0.140	1.293 (0.816–2.049)	0.273	1.489 (0.924–2.400)	0.102
>50	2.025 (1.251–3.277)	0.004	2.264 (1.377–3.725)	0.001	1.915 (1.225–2.994)	0.004	2.201 (1.385–3.497)	0.001
Grade								
I	Reference		Reference		Reference		Reference	
II	1.383 (0.916–2.090)	0.123	1.156 (0.757–1.766)	0.502	1.384 (0.942–2.031)	0.095	1.192 (0.805–1.767)	0.381
III/IV	1.961 (1.272–3.022)	0.002	1.730 (1.113–2.691)	0.015	1.890 (1.261–2.831)	0.002	1.694 (1.122–2.558)	0.012
Unkown	1.475 (0.908–2.395)	0.117	1.369 (0.832–2.252)	0.217	1.424 (0.904–2.243)	0.127	1.317 (0.827–2.097)	0.247
AJCC T stage								
T1a	Reference		Reference		Reference		Reference	
T1b	1.988 (1.382–2.860)	<0.001	1.478 (0.947–2.308)	0.085	1.670 (1.200–2.324)	0.002	1.259 (0.835–1.898)	0.272
T2	3.256 (2.362–4.489)	<0.001	2.781 (1.938–3.990)	<0.001	2.804 (2.105–3.736)	<0.001	2.431 (1.753–3.370)	<0.001
T3/4	4.781 (3.071–7.445)	<0.001	4.273 (2.652–6.884)	<0.001	4.372 (2.927–6.532)	<0.001	4.021 (2.604–6.210)	<0.001
Surgery (major vs. minor resection)	0.959 (0.717–1.283)	0.780	0.974 (0.723–1.312)	0.860	1.121 (0.865–1.452)	0.387	1.180 (0.905–1.539)	0.222
Chemotherapy (yes vs. no)	1.020 (0.823–1.265)	0.856	1.026 (0.809–1.301)	0.832	0.893 (0.728–1.095)	0.275	0.901 (0.720–1.128)	0.364
Radiation (yes vs. no)	0.977 (0.720–1.326)	0.880	0.985 (0.706–1.372)	0.927	0.884 (0.657–1.190)	0.417	0.926 (0.671–1.279)	0.642
LND (yes vs. no)	1.249 (1.010–1.545)	0.040	1.309 (1.053–1.628)	0.015	1.197 (0.981–1.461)	0.076	1.246 (1.017–1.528)	0.034

Abbreviations: AJCC, American Joint Committee on Cancer; CI, confidence interval; CSS, cancer‐specific survival; DSW, divorced/separated/widowed; HR, hazard ratio; LND, lymph node dissection; OS, overall survival; PSM, propensity score matching.

**TABLE 3:** Univariate and multivariate Cox proportional hazard regression analysis of CSS and OS after PSM

Variables	CSS				OS			
	Univariate analysis		Multivariate analysis		Univariate analysis		Multivariate analysis	
	HR (95% CI)	*p* value	HR (95% CI)	*p* value	HR (95% CI)	*p* value	HR (95% CI)	*p* value
Sex (male vs. female)	1.181 (0.993–1.405)	0.060	1.362 (1.132–1.639)	0.001	1.247 (1.058–1.470)	0.008	1.394 (1.170–1.662)	<0.001
Age (year) (>60 vs. ≤60)	0.957 (0.804–1.138)	0.619	1.012 (0.843–1.216)	0.894	1.059 (0.897–1.249)	0.500	1.086 (0.912–1.293)	0.353
Race								
White	Reference		Reference		Reference		Reference	
Asian	0.997 (0.771–1.290)	0.984	1.115 (0.856–1.453)	0.420	0.930 (0.725–1.193)	0.567	1.025 (0.794–1.324)	0.848
Black	1.205 (0.888–1.635)	0.232	1.315 (0.962–1.796)	0.086	1.112 (0.825–1.497)	0.487	1.202 (0.887–1.629)	0.236
Other	0.633 (0.089–4.509)	0.648	0.589 (0.082–4.242)	0.599	0.553 (0.078–3.934)	0.554	0.584 (0.081–4.198)	0.593
Marital status								
Married	Reference		Reference		Reference		Reference	
Single	1.005 (0.784–1.287)	0.972	0.996 (0.774–1.284)	0.978	0.977 (0.771–1.238)	0.845	0.972 (0.763–1.238)	0.816
DSW	1.209 (0.960–1.522)	0.106	1.290 (1.006–1.655)	0.045	1.206 (0.970–1.500)	0.092	1.255 (0.991–1.588)	0.059
Unknown	1.487 (0.989–2.235)	0.056	1.604 (1.060–2.427)	0.025	1.433 (0.968–2.120)	0.072	1.523 (1.023–2.267)	0.059
Tumor size (cm)								
≤20	Reference		Reference		Reference		Reference	
20–50	1.228 (0.817–1.844)	0.323	1.391 (0.916–2.114)	0.122	1.190 (0.819–1.731)	0.362	1.363 (0.927–2.005)	0.115
>50	1.796 (1.212–2.661)	0.004	1.957 (1.305–2.934)	0.001	1.639 (1.141–2.355)	0.008	1.850 (1.272–2.689)	0.001
Grade								
I	Reference		Reference		Reference		Reference	
II	1.338 (0.951–1.882)	0.094	1.154 (0.815–1.633)	0.420	1.259 (0.919–1.724)	0.152	1.109 (0.805–1.528)	0.527
III/IV	1.885 (1.323–2.686)	<0.001	1.688 (1.177–2.421)	0.004	1.716 (1.236–2.383)	0.001	1.556 (1.114–2.175)	0.010
Unkown	1.414 (0.939–2.129)	0.097	1.352 (0.893–2.046)	0.154	1.356 (0.928–1.981)	0.115	1.294 (0.882–1.900)	0.188
AJCC T‐stage								
T1a	Reference		Reference		Reference		Reference	
T1b	1.631 (1.184–2.246)	0.003	1.257 (0.862–1.832)	0.234	1.401 (1.044–1.879)	0.024	1.111 (0.781–1.570)	0.567
T2	2.701 (2.060–3.541)	<0.001	2.340 (1.729–3.167)	<0.001	2.329 (1.825–2.974)	<0.001	2.115 (1.606–2.785)	<0.001
T3/4	3.760 (2.740–5.159)	<0.001	3.182 (2.235–4.530)	<0.001	3.228 (2.414–4.317)	<0.001	2.920 (2.106–4.048)	<0.001
Surgery (major vs. minor resection)	1.150 (0.938–1.410)	0.180	1.082 (0.876–1.336)	0.466	1.219 (1.007–1.474)	0.042	1.199 (0.984–1.461)	0.072
Chemotherapy (yes vs. no)	1.097 (0.921–1.305)	0.299	1.025 (0.844–1.245)	0.802	0.944 (0.800–1.114)	0.495	0.904 (0.752–1.086)	0.282
Radiation (yes vs. no)	1.065 (0.850–1.333)	0.585	1.074 (0.841–1.371)	0.569	0.960 (0.770–1.197)	0.719	1.012 (0.796–1.286)	0.923
LND (yes vs. no)	1.331 (1.106–1.600)	0.002	1.221 (1.006–1.481)	0.043	1.215 (1.022–1.444)	0.027	1.252 (1.046–1.498)	0.014

Abbreviations: AJCC, American Joint Committee on Cancer; CI, confidence interval; CSS, cancer‐specific survival; DSW, divorced/separated/widowed; HR, hazard ratio; LND, lymph node dissection; OS, overall survival; PSM, propensity score matching.

**TABLE 4:** Comparison among the NLND patients and LND patients with lymph node metastasis (LNM+) or without lymph node metastasis (LNM−)

Characteristics	NLND *N* = 376 (%)	LNM (−) *N* = 440 (%)	LNM (+) *N* = 212 (%)	*p* value
Age (year)				0.002
≤60	133 (35.4)	205 (46.6)	100 (47.2)	
>60	243 (64.6)	235 (53.4)	112 (52.8)	
Sex				0.953
Male	176 (46.8)	204 (46.4)	101 (47.6)	
Female	200 (53.2)	236 (53.6)	111 (52.4)	
Race				0.038
White	282 (75.0)	349 (79.3)	174 (82.1)	
Asian	58 (15.4)	48 (10.9)	29 (13.7)	
Black	36 (9.6)	41 (9.3)	9 (4.2)	
Other	0 (0)	2 (0.5)	0 (0.0)	
Marital status				0.469
Married	240 (63.8)	283 (64.3)	127 (59.9)	
Single	51 (13.6)	63 (14.3)	42 (19.8)	
DSW	65 (17.3)	81 (18.4)	32 (15.1)	
Unknown	20 (5.3)	13 (3.0)	11 (5.2)	
Year of diagnosis				0.059
2004–2008	81 (21.5)	85 (19.3)	38 (17.9)	
2009–2013	134 (35.6)	123 (28.0)	68 (32.1)	
2014–2018	161 (42.8)	232 (52.7)	106 (50.0)	
Tumor size (mm)				0.220
≤20	22 (5.8)	38 (8.6)	13 (6.1)	
20–50	156 (41.5)	172 (39.1)	73 (34.4)	
>50	198 (52.7)	230 (52.3)	126 (59.4)	
Grade				0.023
I	39 (10.4)	45 (10.4)	12 (8.8)	
II	187 (49.7)	234 (53.2)	102 (48.1)	
III/IV	102 (27.1)	108 (24.5)	79 (37.3)	
Unknown	48 (12.8)	53 (12.0)	19 (9.0)	
AJCC T stage				<0.001
T1a	111 (29.5)	112 (25.5)	17 (8.0)	
T1b	83 (22.1)	82 (18.6)	27 (12.7)	
T2	153 (40.7)	183 (41.6)	110 (51.9)	
T3/4	29 (7.7)	63 (14.3)	58 (27.4)	
Surgery				0.023
Major resection	73 (19.4)	107 (24.3)	62 (29.2)	
Minor resection	303 (80.6)	333 (75.7)	150 (70.8)	
Chemotherapy				<0.001
Yes	146 (38.8)	227 (51.6)	150 (70.8)	
No	230 (61.2)	213 (48.4)	62 (29.2)	
Radiation				0.003
Yes	48 (12.8)	68 (15.5)	50 (23.6)	
No	328 (87.2)	372 (84.5)	162 (76.4)	

Abbreviations: AJCC, American Joint Committee on Cancer; DSW, divorced/separated/widowed; LND, lymph node dissection; LNM, lymph node metastasis; NLND, non‐LND.

### 3.3 Association of LNM with survival outcomes

**FIGURE 5:**
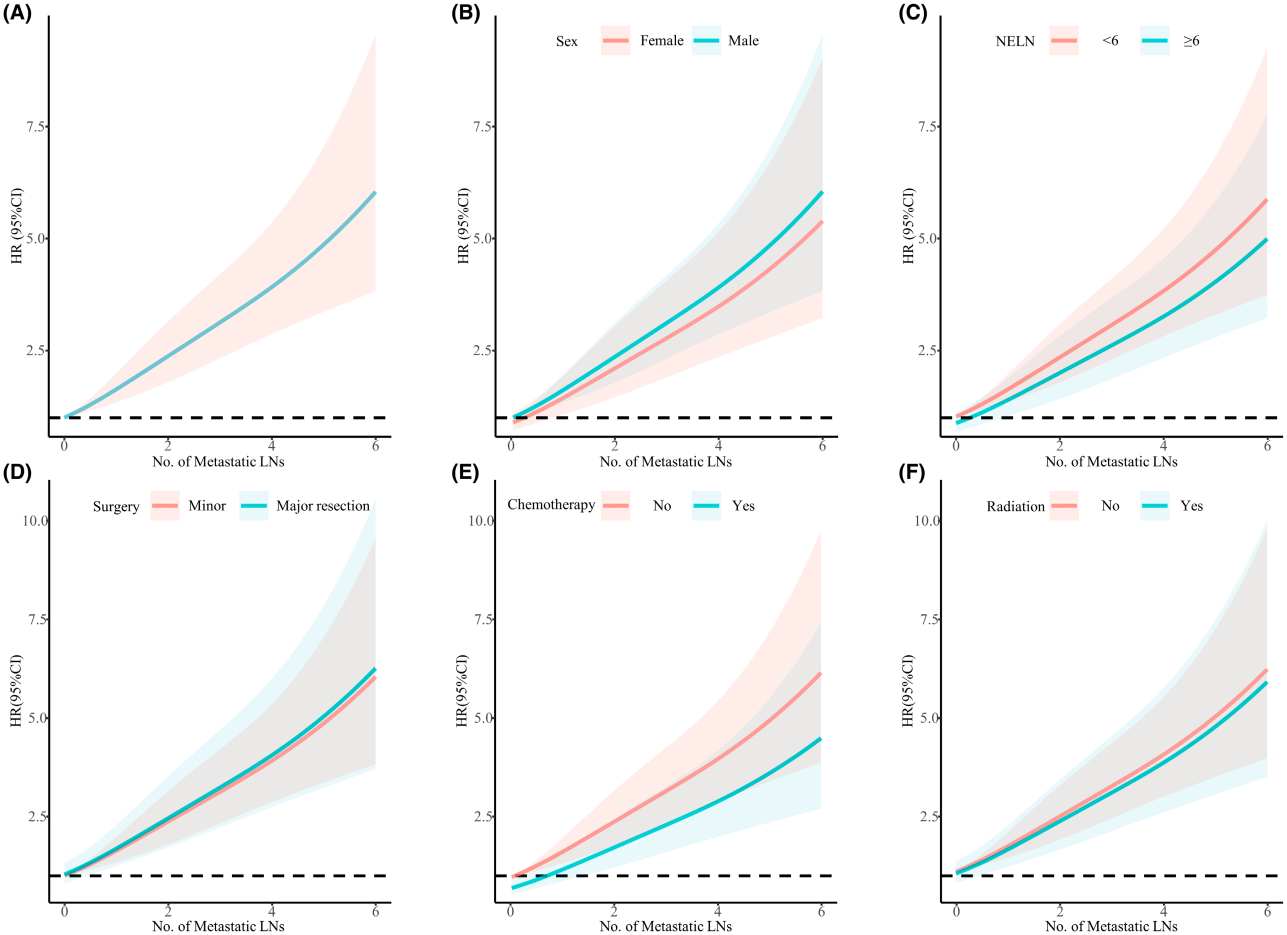
Association between the number of metastatic LNs and CSS in all patients (A) and stratified by sex (B), NELN (C), surgery (D), chemotherapy (E), and radiation (F). The HR was adjusted for age, sex, race, marital status, grade, tumor size, year of diagnosis, AJCC stage, surgery, chemotherapy, and radiation. CI, confidence interval; CSS, cancer‐specific survival; HR, hazard ratio; LNs, lymph nodes; NELN, number of examined lymph nodes; No., number

We estimated the relationship of the number of metastatic LNs with CSS by using Cox proportional hazards models with a 4‐knot RCS. In the smoothed plot of HR versus the number of metastatic LNs, significant effects were observed for the overall association (*p* < 0.001) and the nonlinear association (*p* = 0.024). The trend between the number of metastatic LNs and CSS was consistent when stratified by NELN, sex, the extent of surgery, chemotherapy, and radiation therapy status (Figure 5). Regarding OS, similar results persisted (Figure S4). Then, patients who received LND were categorized as having no nodal metastasis (N0), 1–2 LNM (N1), or ≥3 LNM (N2) according to the number of metastatic LNs. The Kaplan–Meier curves are shown in Figure 3C,D. Patients with N0, N1 and N2 disease had an increasingly worse CSS (median time, N0 50.0 vs. N1 22.0 vs. N2 14.0 months, all *p* < 0.01) and OS (median time, N0 46.0 vs. N1 21.0 vs. N2 14.0 months, all *p* < 0.01). Moreover, after adjusting for confounding factors, N1 (CSS, HR 2.135, 95% CI 1.636–2.788, *p* < 0.001; OS, HR 2.100, 95% CI 1.624–2.717, *p* < 0.001) and N2 (CSS, HR 4.027, 95% CI 2.791–5.811, *p* < 0.001; OS, HR 3.678, 95% CI 2.561–5.282, *p* < 0.001) were independent prognostic factors of both CSS and OS (Figure 3E,F).

### 3.4 Optimal number of LNs examined

**FIGURE 6:**
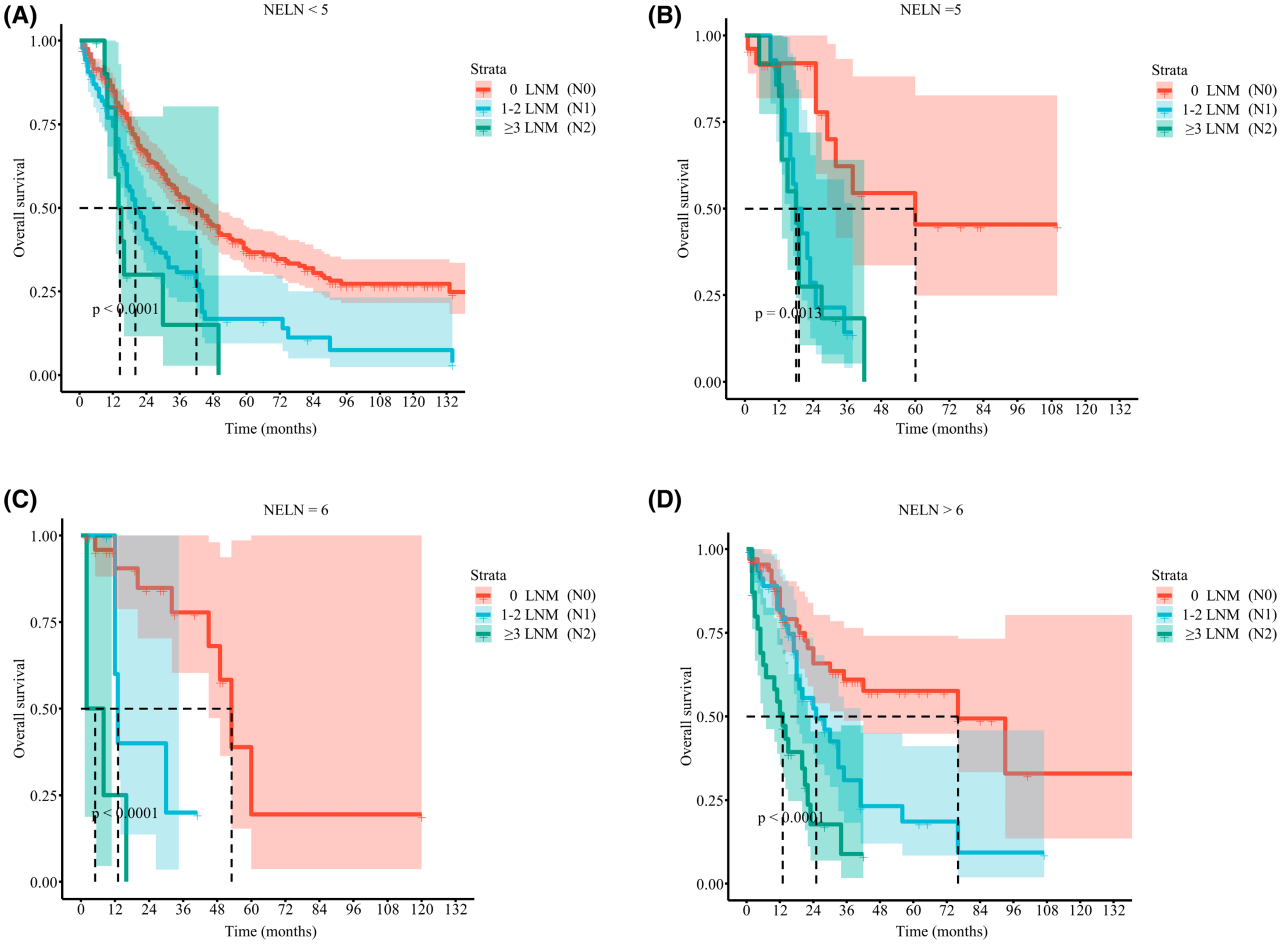
Kaplan–Meier curves of patients with no nodal disease, 1–2 LNM and ≥3 LNM. OS of (A) NELN <5 subgroup. (B) NELN =5 subgroup. (C) NELN =6 subgroup. (D) NELN >6 subgroup. LNM, lymph node metastasis; NELN, number of examined lymph nodes; OS, overall survival

**FIGURE 7:**
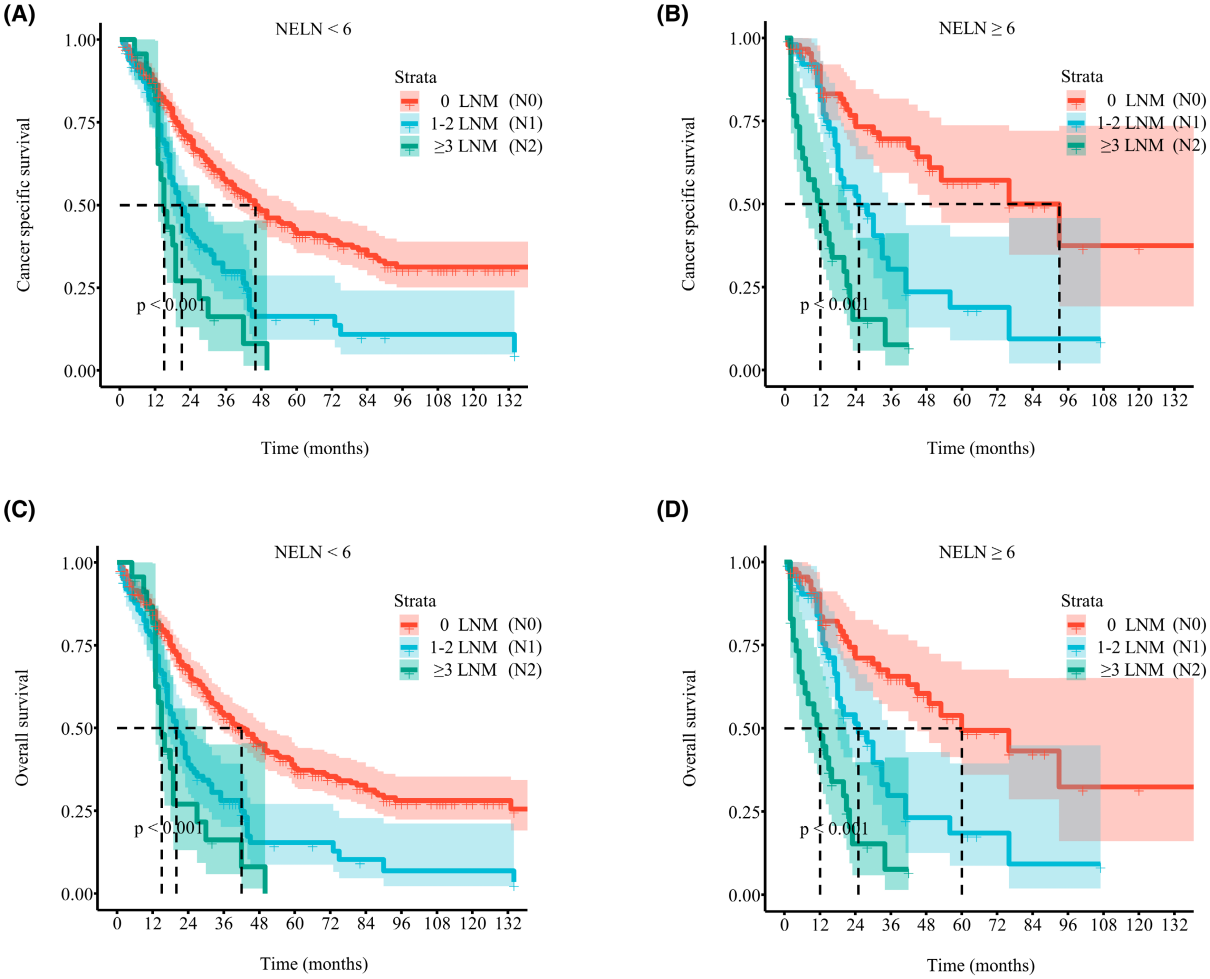
Kaplan–Meier curves of patients with no nodal disease, 1–2 LNM and ≥3 LNM. CSS of (A) NELN <6 subgroup. (B) NELN ≥6 subgroup. OS of (C) NELN <6 subgroup. (D) NELN ≥6 subgroup. CSS, cancer‐specific survival; LNM, lymph node metastasis; NELN, number of examined lymph nodes; OS, overall survival

As the current AJCC staging system recommends a minimum number of six LNs to be harvested for adequate nodal staging. We first validated the optimal cut‐off value of NELN. Among patients who had NELN<5 or NELN =5 (Figure 6A,B), survival outcomes in patients with N1 and N2 disease were no different. Among patients who had NELN =6 or NELN >6, the prognosis was gradually worse as disease was upstaged (Figure 6C,D). The results were the same after data was further merged. For patients with NELN ≥6 (Figure 7B,D), survival outcomes were incrementally poorer as stage rose (median CSS, N0 93.0 vs. N1 25.0 vs. N2 12.0 months; median OS, N0 60.0 vs. N1 25.0 vs. N2 12.0 months, all *p* < 0.01). However, among patients who had NELN <6 (Figure 7A,C), nodal staging failed to stratify CSS (median time, N1 21.0 vs. N2 15.0 months, *p* = 0.073) or OS (median time, N1 20.0 vs. N2 15.0 months, *p* = 0.120) between the N1 and N2 groups. We also found that NELN ≥6 (OR 2.695, 95% CI 1.842–3.943, *p* < 0.001) was an independent risk factor for LNM (Table S1), whereas it was not associated with improved prognosis (*p* > 0.05) (Figure 3E,F). Hence, six LNs is the best cutoff value for LND in the surgical treatment of ICC.

## 4 DISCUSSION

By analyzing a large cohort of more than 1000 resected ICC patients, the present study showed that LND does not seem to improve the outcomes of patients. Among patients who underwent LND, a higher number of LNM was correlated with worse outcomes; thus, LND is essential for optimal staging and prognostic assessment. Moreover, retrieval of at least six LNs is recommended. Relying on the adequacy of LND, the recent arising nodal classification of N0, N1, and N2 has good discrimination to stratify outcomes.

Currently, the value of LND remains a matter of debate in the surgical treatment of ICC. Some studies have indicated that LND does not improve patient survival. Others suggested that it was associated with better long‐term outcomes and should be performed routinely. Tomoaki Yoh et al. analyzed data from three tertiary hepatobiliary centers and found that lymphadenectomy was associated with a better prognosis for patients with node‐negative ICC.^22^ Similarly, in a multicenter retrospective study from Japan, Yuzo Umeda et al. investigated the survival benefit of LND using an inverse probability of treatment weighting procedure, demonstrating that LND during resection of ICC displayed significant therapeutic benefit in improving oncologic outcomes.^15^ Conversely, a recent meta‐analysis by Zhou et al. reported that LND did not alter patient survival regardless of LN status.^23^ Another single‐institution study from Korea retrospectively reviewed data with 17 years of experience and showed no association of LND with OS.^9^ Surprisingly, patients who underwent LND had worse CSS and OS in this study. We speculated that this is possibly due to a higher proportion of LNM in patients who received LND. To test this hypothesis, we divided the LND group into patients with LNM (+) and those without LNM (−) and compared their survival outcomes with those of the NLND group. As expected, we observed that NLND patients showed similar survival times to LNM (−) patients, and only the LNM (+) subgroup was associated with significantly poorer survival. In addition, subgroup analyses confirmed consistent results. To some extent, lymphadenectomy itself may not offer survival benefits. However, this is not a widely adopted practice. Radical surgery plus lymphadenectomy was recommended by expert consensus and National Comprehensive Cancer Network (NCCN) guidelines. From the perspective of surgical oncology, LND is crucial to guarantee oncological radicality. Notably, the major advantage of LND is to provide information about nodal staging and to inform prognosis, as the diagnostic accuracy of preoperative imaging identification of LNM is limited.^24^, ^25^ However, not all patients had LNM. Therefore, developing preoperative noninvasive tools to precisely predict LN status is paramount.

In general, the presence of regional LNM often portends a poor prognosis.^12^, ^26^, ^27^, ^28^ A growing number of studies have shown that the number of metastatic LNs has an impact on prognosis in many cancers.^29^, ^30^ However, there remains insufficient data regarding their association in ICC. Herein, we used RCS curves to offer a visual association between the number of metastatic LNs and prognosis. The data showed significantly higher mortality risks with an increasing metastatic LNs. As the latest AJCC‐staging system only defined the N‐staging based on the status of LNs but not on the number of LNM,^21^ a consensus statement regarding reasonable nodal staging is still not available. In a single‐center retrospective study on 87 patients with ICC, Sung Hyun Kim et al. divided patients into three groups and compared their survival outcomes according to the metastatic LN number: N0: no metastasis; N+ <4: 1 to 3 metastatic LNs; N+ ≥4: 4 or more metastatic LNs. Their data showed that the three groups had significant differences in survival.^14^ Recently, Zhang et al. proposed a novel nodal staging of N0, N1 (1–2 LNM), and N2 (≥3 LNM) using a large multi‐institutional database of patients with curative‐intent resection ICC, and this nodal staging could well stratify outcomes of patients.^17^ When more than six or more LNs were examined, we also observed that patients with no nodal metastasis, 1–2 LNM, and ≥3 LNM had a monotonically increasing chance of death. Furthermore, a similar finding was reported in a few studies in which the involvement of three or more LNs was a significant risk factor for poor prognosis.^27^, ^31^, ^32^ Taken together, the above evidence supported that the nodal staging classifications of ICC should be further stratified based on the number of metastatic LNs. Consistent with the eighth edition of AJCC recommendations,^21^ in this study, the novel nodal classification could not stratify prognosis between the N1 and N2 groups among patients who had NELN <6, indicating that adequate pathologic staging can only be obtained by the retrieval of at least six LNs. Of note, NELN ≥6 was an independent risk factor for LNM in our data. This reflects the inadequate number of LNs sampled in the study population. Accordingly, we strongly suggest a routine LND of six or more LNs to increase the detection rates of LNM during liver resection for ICC. This may have clinical implications for better prognostic stratification.

To the best of our knowledge, this is a population‐based real‐world study using large sample sizes with rigorous statistical analysis on such issues. Nonetheless, there were some limitations to our study. Although PSM in addition to conventional multivariate analysis were conducted, the nature of the retrospective database led to intrinsic selection bias. Patients with higher probability of being node‐positive, or more advanced‐stage disease are prone to receive LND. In addition, the SEER database itself does not provide information about the detailed extent of lymphadenectomy, preoperative imaging data, physical condition, and molecular alterations of tumors, which could influence the treatment selection and the survival outcome.^33^ Lastly, future well‐designed prospective clinical trials are needed to validate the broad applicability of the study findings.

## 5 CONCLUSIONS

This population‐based study reveals that the LND failed to show survival benefit in patients with ICC. A significant positive association between the number of LNM and poor outcomes was observed. LND with at least 6 LNs harvested plays an important role for accurate nodal staging. With adequate LND, the recently proposed nodal classification of N0, N1, and N2 is of considerable clinical significance in the prognostic stratification of ICC patients.

## AUTHOR CONTRIBUTIONS

**Jiang Zhu:** Conceptualization (equal); data curation (equal); formal analysis (equal); funding acquisition (equal); investigation (equal); project administration (equal); resources (equal); software (equal); supervision (equal); validation (equal); visualization (equal); writing – original draft (equal); writing – review and editing (equal). **Chang Liu:** Formal analysis (equal); software (equal); writing – review and editing (equal). **Hui Li:** Data curation (equal). **Haoyu Ren:** Data curation (equal). **Yunshi Cai:** Investigation (equal). **Tian Lan:** Methodology (equal). **Hong Wu:** Conceptualization (equal); project administration (equal); resources (equal); software (equal); visualization (equal); writing – original draft (equal); writing – review and editing (equal).

## FUNDING INFORMATION

This work was supported by the National Natural Science Foundation of China (82173124, 82103533, 81972747, and CSTB2022NSCQ‐MSX0477 ), the China Postdoctoral Science Foundation (2022TQ0393).

## CONFLICT OF INTEREST

The authors declare that they have no competing interests.

## ETHICS APPROVAL AND CONSENT TO PARTICIPATE

Ethical approval and written informed consent were not needed, as the study is based on anonymized publicly available data.

## Supporting information


Figure S1.

Figure S2.

Figure S3.

Figure S4.

Table S1.

## Data Availability

The datasets used during the current study are available from the corresponding author on reasonable request.
