# Supplementary figures and images for: Adequate lymph node dissection is essential for accurate nodal staging in intrahepatic cholangiocarcinoma: A population‐based study
Source: Cancer Med. 2023 Jan 16;12(7):8184–98. doi: 10.1002/cam4.5620 (PMC10134328; doi:10.1002/cam4.5620)

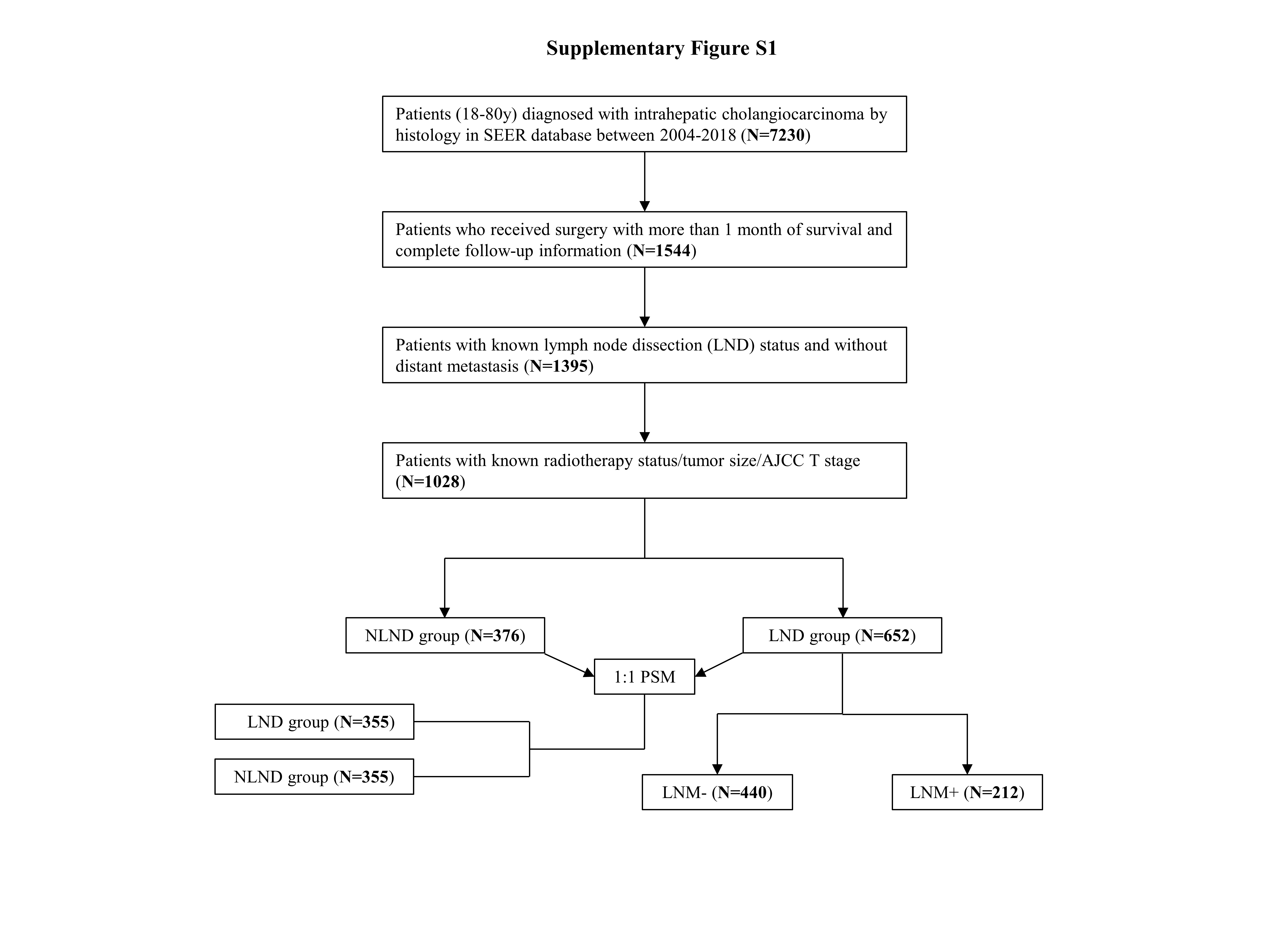

Supplement: Supplementary file 1 — Figure S1. [file CAM4-12-8184-s003.tif]

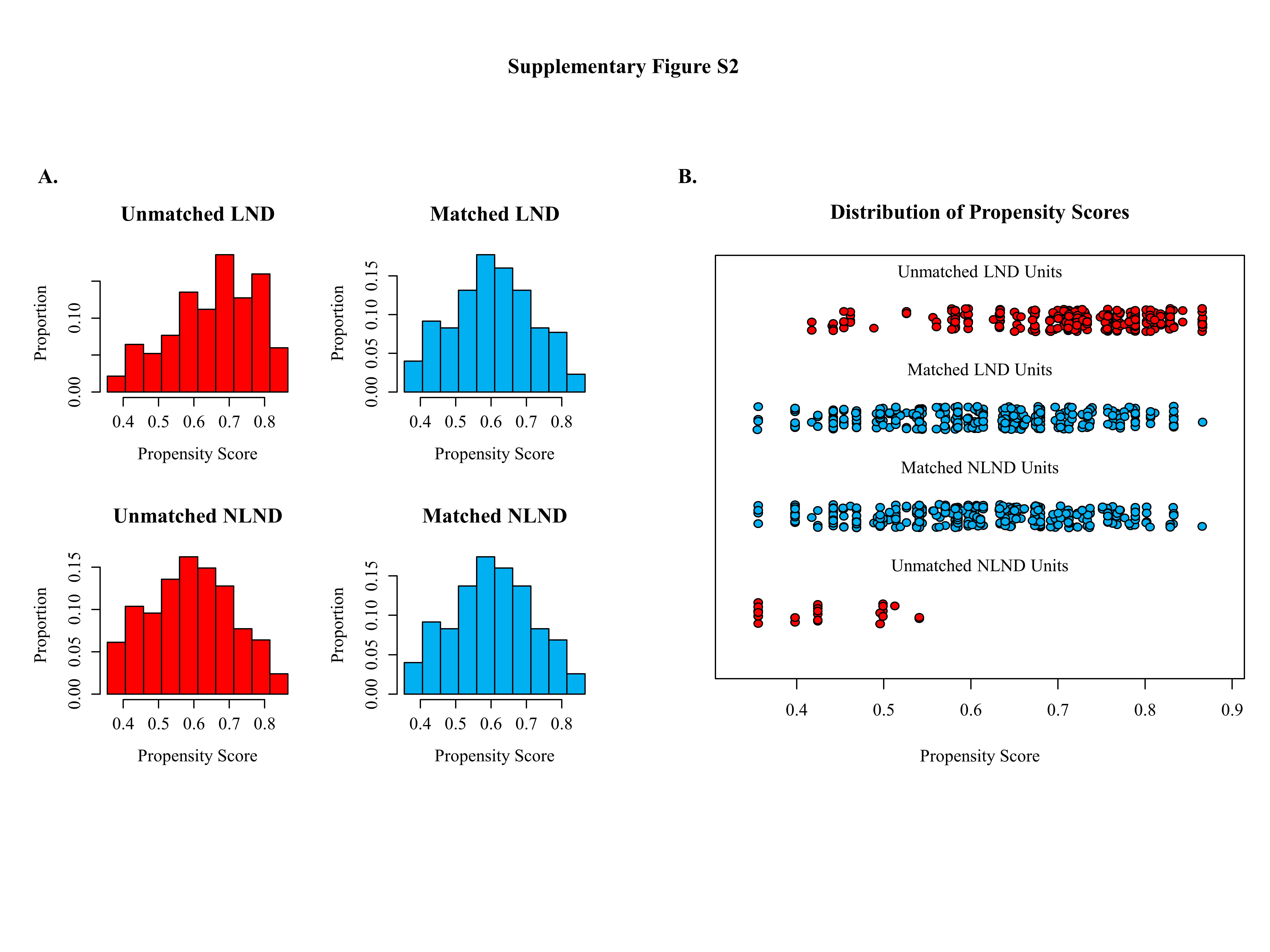

Supplement: Supplementary file 2 — Figure S2. [file CAM4-12-8184-s002.tif]

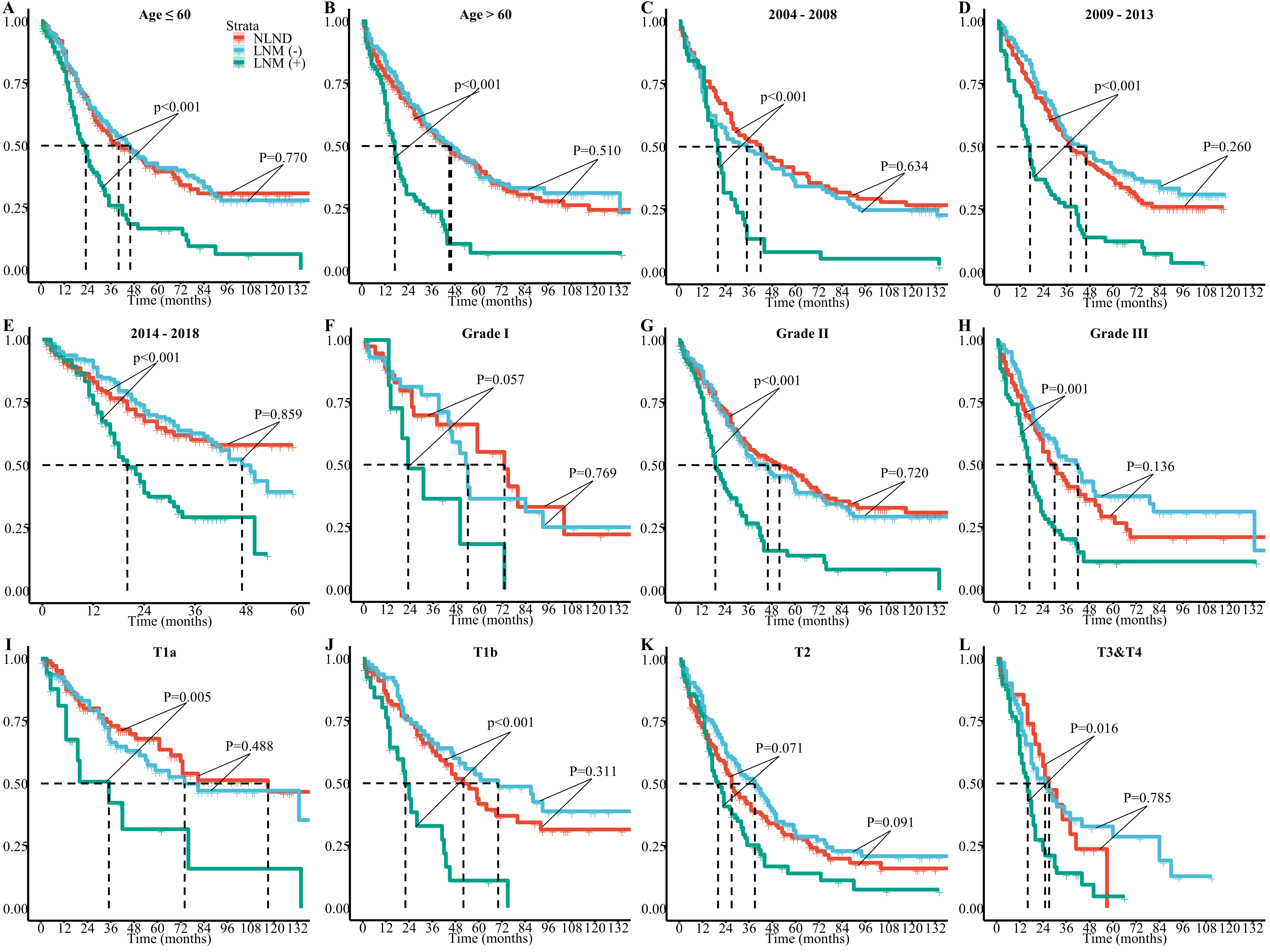

Supplement: Supplementary file 3 — Figure S3. [file CAM4-12-8184-s001.tif]

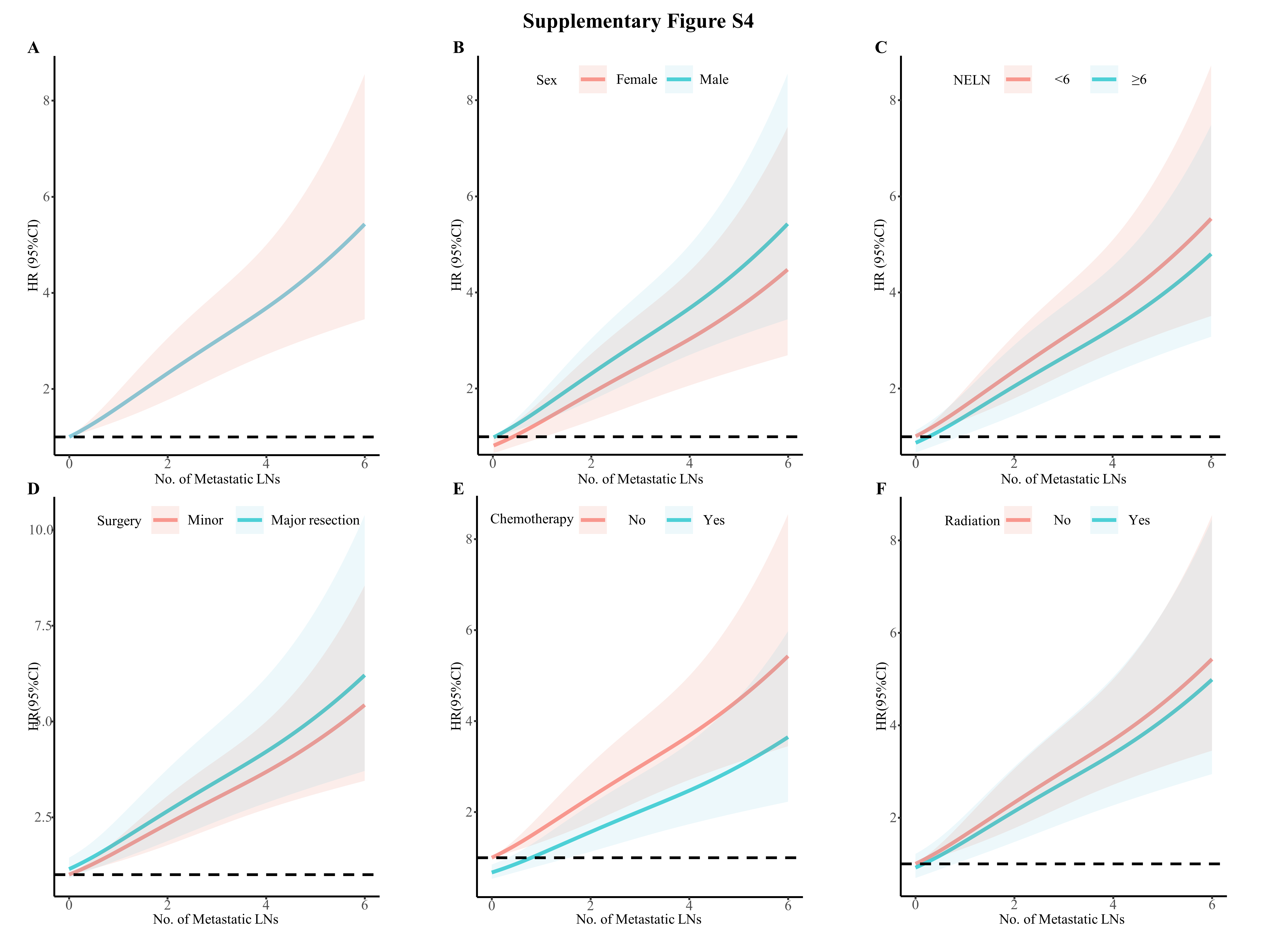

Supplement: Supplementary file 4 — Figure S4. [file CAM4-12-8184-s004.tif]
