# Supplementary material for: Adequate lymph node dissection is essential for accurate nodal staging in intrahepatic cholangiocarcinoma: A population‐based study
Source: Cancer Med. 2023 Jan 16;12(7):8184–98. doi: 10.1002/cam4.5620 (PMC10134328; doi:10.1002/cam4.5620)
Supplement: Supplementary file 5 — Table S1. [file CAM4-12-8184-s005.docx]

**Supplementary materials**

**Supplementary Table S1**. Univariate and multivariate logistic regression analysis of factors for lymph node metastasis.in patients with LND.

| Variables | Univariate analysis | | Multivariate analysis | |  |
| --- | --- | --- | --- | --- | --- |
|  | OR (95%CI) | *P* value | OR (95%CI) | *P* value |  |
| Sex (Male vs. Female) | 1.053 (0.758-1.462) | 0.759 | 1.080 (0.755-1.546) | 0.673 |  |
| Age (y) (>60 vs. ≤60) | 0.977 (0.704-1.357) | 0.890 | 0.963 (0.674-1.378) | 0.839 |  |
| Number of nodes harvested  (≥6 vs. <6) | 2.684 (1.879-3.836) | <0.001 | 2.695 (1.842-3.943) | <0.001 |  |
| Race |  |  |  |  |  |
| White | Reference |  | Reference |  |  |
| Asian | 1.212 (0.738-1.989) | 0.447 | 1.140 (0.672-1.934) | 0.628 |  |
| Black | 0.440 (0.209-0.927) | 0.031 | 0.043 (0.203-0.964) | 0.040 |  |
| Tumor size (cm) |  |  |  |  |  |
| ≤20 | Reference |  | Reference |  |  |
| 20 - 50 | 1.241 (0.624-2.465) | 0.538 | 1.302 (0.619-2.741) | 0.487 |  |
| >50 | 1.601 (0.823-3.117) | 0.166 | 1.311 (0.613-2.804) | 0.486 |  |
| Grade |  |  |  |  |  |
| I | Reference |  | Reference |  |  |
| II | 1.635 (0.830-3.220) | 0.155 | 1.363 (0.650-2.855) | 0.412 |  |
| III/IV | 2.743 (1.362-5.523) | 0.005 | 1.820 (0.842-3.933) | 0.128 |  |
| Unkown | 1.344 (0.589-3.067) | 0.482 | 1.144 (0.470-2.783) | 0.767 |  |
| AJCC T stage |  |  |  |  |  |
| T1a | Reference |  | Reference |  |  |
| T1b | 2.169 (1.110-4.241) | 0.024 | 1.952 (0.878-4.338) | 0.101 |  |
| T2 | 3.960 (2.257-6.949) | <0.001 | 3.482 (1.849-6.558) | <0.001 |  |
| T3/4 | 6.065 (3.255-11.303) | <0.001 | 5.360 (2.699-10.645) | <0.001 |  |

Abbreviations: OR, odds ratio; CI, confidence interval.
